# Development and Pilot-Testing of an Optimized Conversational Agent or “Chatbot” for Peruvian Adolescents Living With HIV to Facilitate Mental Health Screening, Education, Self-Help, and Linkage to Care: Protocol for a Mixed Methods, Community-Engaged Study

**DOI:** 10.2196/55559

**Published:** 2024-05-07

**Authors:** Jerome T Galea, Diego H Vasquez, Neil Rupani, Moya B Gordon, Milagros Tapia, Karah Y Greene, Lenka Kolevic, Molly F Franke, Carmen Contreras

**Affiliations:** 1 School of Social Work College of Behavioral and Community Sciences University of South Florida Tampa, FL United States; 2 Department of Global and Social Medicine Harvard Medical School Boston, MA United States; 3 Socios En Salud Sucursal Lima Peru; 4 Morsani College of Medicine University of South Florida Tampa, FL United States; 5 Instituto Nacional de Salud del Niño Lima Peru; 6 Departamento Académico de Pediatría Universidad Nacional Mayor de San Marcos Lima Peru; 7 Harvard Global Health Institute Harvard University Cambridge, MA United States

**Keywords:** chatbot, digital assistant, depression, HIV, adolescents

## Abstract

**Background:**

Adolescents living with HIV are disproportionally affected by depression, which worsens antiretroviral therapy adherence, increases viral load, and doubles the risk of mortality. Because most adolescents living with HIV live in low- and middle-income countries, few receive depression treatment due to a lack of mental health services and specialists in low-resource settings. Chatbot technology, used increasingly in health service delivery, is a promising approach for delivering low-intensity depression care to adolescents living with HIV in resource-constrained settings.

**Objective:**

The goal of this study is to develop and pilot-test for the feasibility and acceptability of a prototype, optimized conversational agent (chatbot) to provide mental health education, self-help skills, and care linkage for adolescents living with HIV.

**Methods:**

Chatbot development comprises 3 phases conducted over 2 years. In the first phase (year 1), formative research will be conducted to understand the views, opinions, and preferences of up to 48 youths aged 10-19 years (6 focus groups of up to 8 adolescents living with HIV per group), their caregivers (5 in-depth interviews), and HIV program personnel (5 in-depth interviews) regarding depression among adolescents living with HIV. We will also investigate the perceived acceptability of a mental health chatbot, including barriers and facilitators to accessing and using a chatbot for depression care by adolescents living with HIV. In the second phase (year 1), we will iteratively program a chatbot using the SmartBot360 software with successive versions (0.1, 0.2, and 0.3), meeting regularly with a Youth Advisory Board comprised of adolescents living with HIV who will guide and inform the chatbot development and content to arrive at a prototype version (version 1.0) for pilot-testing. In the third phase (year 2), we will pilot-test the prototype chatbot among 50 adolescents living with HIV naïve to its development. Participants will interact with the chatbot for up to 2 weeks, and data will be collected on the acceptability of the chatbot-delivered depression education and self-help strategies, depression knowledge changes, and intention to seek care linkage.

**Results:**

The study was awarded in April 2022, received institutional review board approval in November 2022, received funding in December 2022, and commenced recruitment in March 2023. By the completion of study phases 1 and 2, we expect our chatbot to incorporate key needs and preferences gathered from focus groups and interviews to develop the chatbot. By the completion of study phase 3, we will have assessed the feasibility and acceptability of the prototype chatbot. Study phase 3 began in April 2024. Final results are expected by January 2025 and published thereafter.

**Conclusions:**

The study will produce a prototype mental health chatbot developed with and for adolescents living with HIV that will be ready for efficacy testing in a subsequent, larger study.

**International Registered Report Identifier (IRRID):**

DERR1-10.2196/55559

## Introduction

At the end of 2022, among the global population of children younger than 15 years of age, approximately 1.5 million were living with HIV, 130,000 new cases of HIV were reported, and 84,000 died from HIV-related causes [1]. AIDS remains the second leading cause of death among adolescents globally [2]. Lower adherence to antiretroviral therapy (ART) is the primary cause of AIDS mortality among people living with HIV across the life span; however, relative to children and adults, adolescents living with HIV are less likely to achieve viral suppression, a precursor to HIV treatment failure [3,4]. While many factors negatively affect ART adherence, depression disproportionately affects adolescents living with HIV compared to other age groups [5-7] and is associated with worse HIV treatment outcomes [8,9]. If left untreated, adolescents living with HIV and depression often face mounting problems, including poorer quality of life, more rapid progression of HIV, and premature death [10,11]. Moreover, depression complicates the transition from pediatric to adult HIV care in which adolescents living with HIV already face barriers to care retention, reduced ART adherence, lower CD4 lymphocyte counts, and lower viral suppression rates [3,8].

Despite the disproportionately negative impact of depression on adolescents living with HIV, a recent review of mental health interventions for adolescents with (or at risk of) HIV concluded that “surprisingly little” is known about treatments for this population [12]. Especially scarce are low-cost, evidence-based mental health treatments that could be easily scaled in resource-limited settings [13], including much of Latin America and the Caribbean, home to approximately 42,000 youths aged younger than 15 years living with HIV and where, in 2022, there were approximately 5300 new HIV infections and 3570 deaths among this population [14]. Emerging research has begun to demonstrate the benefit of treating comorbid depression and HIV [15,16], especially among adolescents [12]. Increasingly emphasized are integrated care models that simultaneously treat both HIV and depression to achieve better outcomes at the individual and programmatic levels for both morbidities [17-20]. The integration of mental health services into common priority health care platforms, including HIV, is part of a broader movement to increase access to mental health services for all people [21]. However, for young people, existing literature largely focuses on pathways to care for severe mental illnesses, leaving a gap in knowledge for other mental health conditions [22]. The scarcity of mental health resources is further exacerbated in low- and middle-income countries, where interventions are often reserved for only the most severe cases.

In Lima, Peru, we found evidence that depression care for adolescents living with HIV is an unmet health need unless symptoms are severe (eg, depression with psychosis and suicidal ideation) [23]. At the same time, we also observed in a small pilot study that the majority (92%) of adolescents living with HIV with depression did not have severe symptoms [24]. Adolescents living with HIV with mild to moderate depression do not receive support services (education, self-help strategies to develop coping skills and community bonding, and low-intensity care) as part of routine care that could help cushion the escalation to more severe depression and thereby sustain their commitment to HIV care. As a result, these young people may miss an early opportunity to attenuate depressive symptoms and their potential impact on adherence to ART, resulting in suboptimal individual and programmatic outcomes.

To address this gap, this project aims to use chatbot technology to provide education, self-help, and care linkage for adolescents living with HIV and depression. Chatbots—conversational agents that use text or voice in a human-like way to deliver information—allow users to receive information through multiple existing platforms such as SMS text messages, websites, WhatsApp, and Facebook Messenger without users needing special software and are ideal for low-resource environments. Furthermore, chatbots are already widely used in consumer environments due to their ability to quickly provide personalized information and increase the probability of purchase. In the health sector, the use of chatbots is less frequent but has been applied to providing mental health interventions [25,26] and, more recently, to link people with information and testing related to COVID-19 [27]. In Peru, during the COVID-19 pandemic, we observed that adolescents living with HIV had mobile devices and were able and amenable to receiving health information using technology [28]. In addition, the mental health team at Socios En Salud Sucursal Peru (the performance site for this study) developed and deployed mental health chatbots to screen for depression among adults during the first year of COVID-19, reaching >40,000 people in the country [29,30]. Building on our experience with adolescents living with HIV, mental health, and chatbot technology, we aim to create a novel chatbot dedicated to the unique mental health needs of adolescents living with HIV.

This project aims first to develop a chatbot, optimized to provide education, self-help skills, and care linkage for depression in adolescents living with HIV. Once a prototype chatbot is programmed, it will then be tested for feasibility and acceptability among adolescents living with HIV in Peru. We hypothesize that the chatbot will be feasible and acceptable to adolescents living with HIV to access depression education, self-help skills, and care linkage.

## Methods

### Study Overview

**Figure 1:**
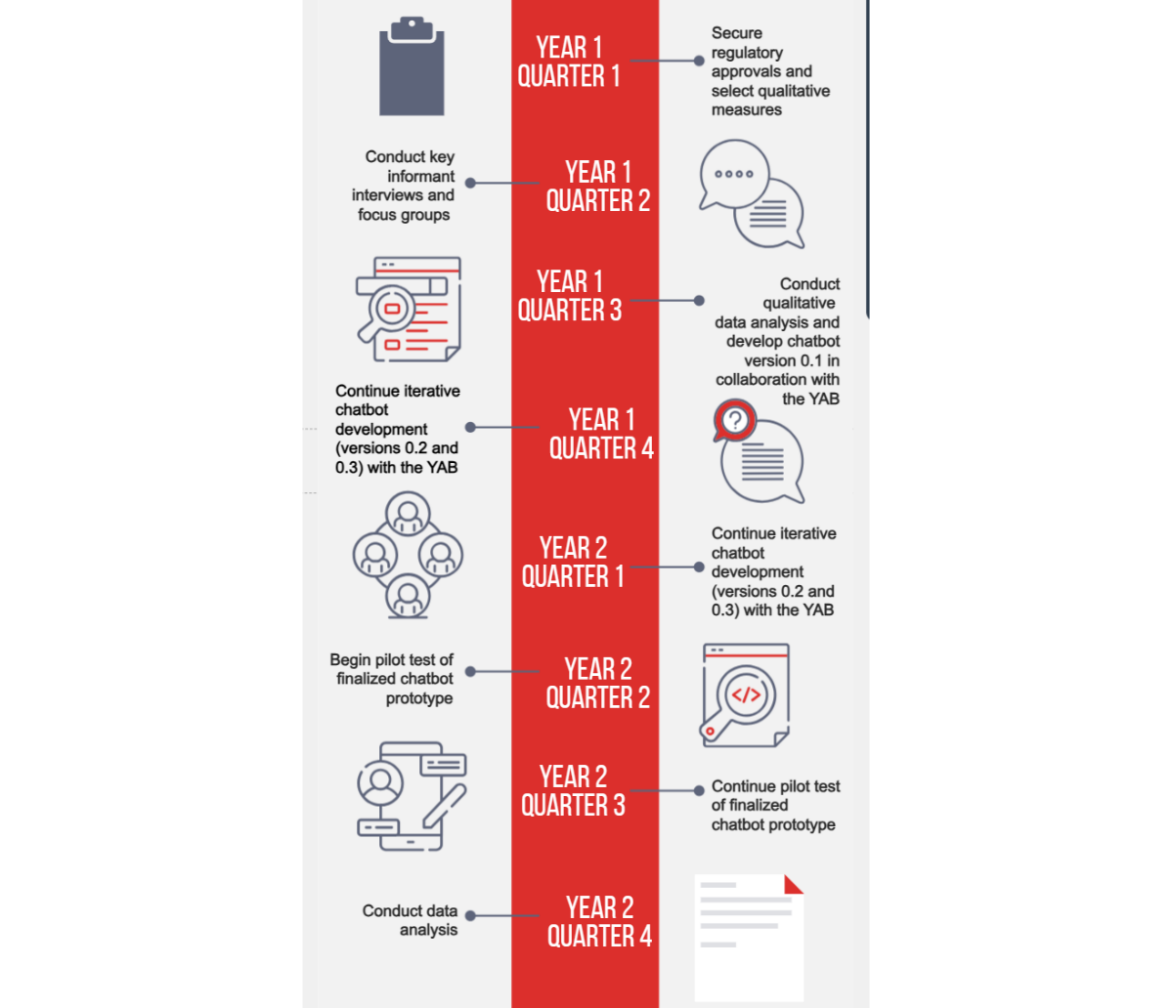
Study aims and process for the development and pilot-testing of an optimized conversational agent or “chatbot” for Peruvian adolescents living with HIV to facilitate mental health screening, education, self-help, and linkage to care. YAB: Youth Advisory Board.

Using a mixed method, qualitative-quantitative design, we will develop (study phases 1 and 2) and pilot-test (study phase 3) a chatbot to deliver depression education, self-help skills, and care linkage for Peruvian adolescents living with HIV. All research instruments and the design of the chatbot will be done in collaboration with a Youth Advisory Board (YAB) comprised of adolescents living with HIV in Peru. Chatbot development will follow a human-centered approach, whereby feedback from the YAB (potential users of a future mental health chatbot) is solicited early and repeatedly during the development process to minimize design errors and maximize the fit of the chatbot to the preferences of the target population before pilot-testing. Figure 1 displays the study timeline and key milestones.

### Study Population

Adolescent participants will be youths aged 10-19 years living with HIV in Lima, Peru. We will purposely recruit a diverse population of participants, including male and female participants, adolescents who acquired HIV at or near birth and adolescents who acquired HIV recently in life, pregnant adolescents, sexual and gender minorities (identifying as transgender, gay, or bisexual), and adolescents who have lost parents to HIV. Adult participants (caregivers and HIV care personnel) will be 18 years of age or older. Caregivers may be the adolescent’s parents, family members, or other legal guardians providing care to an adolescent living with HIV. HIV care personnel will be persons currently working in the Peruvian National HIV care system with adolescents aged 10-19 years and living with HIV.

### Youth Advisory Board

We will invite up to 10 adolescents aged 10-19 years living with HIV and residing in Lima, Peru to participate in the YAB. To ensure a heterogeneous group in terms of sex, sexual orientation, age, and HIV acquisition type, the adolescents will be recruited in collaboration with the infectious disease department of Peru’s Instituto Nacional de Salud del Niño (National Institute of Children’s Health), which cares for children and adolescents up to 18 years of age. Additionally, up to 2 adolescents between 18 and 19 years of age will be recruited through other national services of care for adults living with HIV. The YAB will review and provide feedback on all research documents (consents and instruments) and multiple versions of the chatbot while in the development phase (study phase 1).

### Ethical Considerations

All study procedures have been approved by the Comité Institucional de Bioética de VÍA LIBRE, which is an institutional review board (IRB) of record for the performance site, Socios En Salud Sucursal, Peru. The IRB at the University of South Florida formally agreed to rely on VÍA LIBRE for the review, approval, and continuing oversight of the research project under an interagency IRB Authorization Agreement (University of South Florida IRB study # 005124). For study phases 1 and 2, the data are deidentified, and any participant names or other identifying information will be deleted from interview transcripts; only a study ID will be used for identification purposes. For phase 3, participant names and phone numbers will be stored on a password-protected computer only accessible to the study staff in Peru, and participants will be identified by a study ID for research purposes. All study participants (phases 1-3) and YAB members (for each meeting attended) will be compensated PEN 100 (approximately US $26.50) in vouchers redeemable at multiple local grocery stores. Transportation will be provided for in-person study visits.

### Development of a Prototype Chatbot to Provide Depression Education, Self-Help, and Care Linkage

#### Phase 1: Formative Data Collection to Inform Chatbot Development

##### Overview

We will conduct up to 6 focus groups: 3 with adolescents aged 10-14 years living with HIV and 3 with adolescents aged 15-19 years living with HIV and residing in Lima, Peru. Additionally, up to 10 interviews will be conducted with key informants: 5 with caregivers of adolescents living with HIV and 5 with HIV care personnel. A semistructured interview guide for the focus groups and key informant interviews will be used to identify thoughts, views, and consequences of depression in adolescents living with HIV. Participants will also be asked about their perceived acceptability of the proposed mental health chatbot for adolescents living with HIV using open-ended questions derived from 7 core intervention acceptability constructs: affective attitude, ethics, burden, coherence of intervention, opportunity costs, perceived effectiveness, and self-efficacy [31]. Focus groups and key informant interviews will be audio recorded, transcribed verbatim, and analyzed using Dedoose qualitative data analysis software (version 9.2.007; SocioCultural Research Consultants, LLC).

##### Eligibility Inclusion Criteria

The inclusion criteria are adolescents who are older than 10 years and younger than 19 years of age, living with HIV, and aware of HIV diagnosis; caregivers who are 18 years and older and currently caring for an adolescent living with HIV; and HIV care personnel who are 18 years and older and currently working in the Peruvian National HIV care system with adolescents aged 10-19 years and living with HIV.

##### Eligibility Exclusion Criteria: Adolescents, Caregivers, and HIV Care Personnel

The exclusion criterion is any acute condition (emotional, physical, and social) that, by the decision of the investigator, could place the participant at significant risk due to participation in the study.

#### Data Analysis

Qualitative data will be analyzed using the framework analysis approach [32], beginning with a preliminary codebook derived from the focus group and key informant interview guides. As coding progresses, de novo codes will be added for emergent themes. After all transcripts are coded, reports that contain all text segments for each code will be compiled and analyzed for themes within each code. Emergent themes will be reported using illustrative quotes. The COREQ (Consolidated Criteria for Reporting Qualitative Data) checklist [33] will be completed to enhance data rigor and methodological transparency.

#### Phase 2: Chatbot Design and Programming With Iterative YAB Feedback

We will program the chatbot using the platform SmartBot360 [34]. The YAB will participate in ongoing or iterative testing of the chatbot over several months. First, we will develop an initial version of the chatbot with basic functionality (version 0.1) and convene the YAB to provide early feedback and suggestions, which will be incorporated into the chatbot design and programming yielding version 0.2; the process of YAB feedback solicitation and feedback incorporation will be repeated to arrive at version 0.3. During the YAB consultations, in addition to feedback on the chatbot functionality (eg, presentation, navigation, and menus), the YAB will guide the research team on content for up to 6 educational videos, or graphics and animations on depression and coping skills (30-90 seconds long) to be delivered by the chatbot. Once the YAB reviews and approves version 0.3, we will finalize the remaining programming suggestions to arrive at the pilot version of the chatbot, 1.0, for feasibility and acceptability testing.

### Assessing the Feasibility and Acceptability of the Chatbot Prototype

#### Phase 3: Pilot-Testing for Feasibility and Acceptability of Chatbot Prototype Version 1.0

We will recruit up to 50 adolescents living with HIV naïve to the development phase to test chatbot version 1.0 for feasibility and acceptability. Participants first will complete a survey to collect information on sociodemographics (age, sex, sexual and gender identity, and education level), HIV (HIV acquisition route, current viral load, and frequency of missed HIV care visits), knowledge and history of depression, and previous chatbot use (see Table 1 for all planned measures). Next, participants will interact with the chatbot and then complete a second questionnaire to assess their acquired knowledge about depression and measure the acceptability and feasibility of the chatbot. The acceptability and feasibility questionnaires will include 3 measures: the Acceptability of the Intervention Measure, the Intervention Appropriateness Measure, and the Feasibility of Intervention Measure [35]. Finally, participants will be offered the opportunity to use the chatbot on their own for 2 additional weeks and will receive a follow-up SMS text message to rate their experience. The number of adolescents living with HIV who use the chatbot on their own and the number of acceptances and refusals to use the chatbot will be recorded during these 2 weeks as further measures of acceptability.

**Table 1:** Planned pilot-testing survey measures, administration point, target, and rationale to assess the feasibility and acceptability of an optimized conversational agent or “chatbot” among 50 adolescents living with HIV in Peru to facilitate mental health screening, education, self-help, and linkage to care.

Administration point and measure or instrument		Target	Rationale
**Before chatbot use**			
	Sociodemographic	Participant characteristics	Describe the study population including HIV or health information and previous chatbot use
	Adolescent Depression Knowledge Questionnaire (ADKQ) [36]	Current understanding of depression	Measure baseline depression knowledge prior to interacting with the chatbot
	Patient Health Questionnaire-9 adolescent version [37]	Current depressive symptoms	Measure baseline presence of depression symptoms
	General Anxiety Disorder-7 questionnaire [38]	Current anxiety symptoms	Measure baseline presence of anxiety symptoms
	The Self-Stigma of Seeking Help Scale [39]	Label avoidance	Aid in providing appropriate services based on stigma measure
	Attitudes Towards Mental Health Treatment [40]	Feelings about getting mental health help	Aid in providing appropriate services based on attitudes toward treatment
	Perceived Stress Scale [41]	Current stress levels	Aid in providing appropriate services based on stress levels
**After chatbot use**			
	ADKQ [36]	Current understanding of depression	Measure change of depression knowledge after interacting with the chatbot
	Acceptability of Intervention Measure [35]	Acceptability of the chatbot	Measures the perception of adolescents living with HIV that the chatbot is agreeable, palatable, or satisfactory
	Intervention Appropriateness Measure [35]	Appropriateness of the chatbot	Measures the perceived fit, relevance, or compatibility of the chatbot to address depression among adolescents living with HIV
	Feasibility of Intervention Measure [35]	Feasibility of the chatbot	Measures the extent to which the chatbot can be successfully used
	Acceptability of mental health information delivered by the chatbot	Acceptability (or perceived acceptability) of mental health information delivered by chatbot and perceived continued use of chatbot	Measures the extent to which the user believes they would use the mental health information delivered by the chatbot and use the chatbot in the future and recommend to others
	Chatbot feature satisfaction scale	Satisfaction with core chatbot features	Measures the extent to which chatbot features (eg, graphics, information, and self-help tools) are liked

#### Data Analysis

Data will be cleaned, and summary tables will be generated. Due to the small sample size, tests of association are not planned, and the main analysis is limited to descriptive statistics. For the Adolescent Depression Knowledge Questionnaire, which is applied before and again after interacting with the chatbot (during a single study visit), we will use matched paired 1-tailed *t* tests. Using G*Power (version 3.1; Heinrich Heine University Dusseldorf) [42,43], with a medium effect size, a sample size of 45 would be sufficient to detect effects if they are present using a matched pair 1-tailed *t* test.

## Results

The study was awarded in April 2022, approved by IRB VÍA LIBRE in November 2022, received funding in December 2022, and commenced recruitment in March 2023. Study phases 1 and 2 are complete as of January 2024, and study phase 3 began in April 2024. Results on the feasibility and acceptability of the chatbot are expected by January 2025 and published thereafter.

## Discussion

This study tests a pragmatic, inexpensive, adaptable, and highly scalable solution to increase access to a range of depression care services for adolescents living with HIV, beginning with education and self-help skills. Given the global shortage of mental health professionals to deliver depression care, especially in low- and middle-income countries where >90% of people with HIV live [44], we anticipate that our mental health chatbot holds the potential to go beyond depression identification and care referral; it could empower adolescents living with HIV by providing depression education and practical self-help coping skill. Moreover, the chatbot may be especially attractive to adolescents not wanting to immediately speak with another person due to the stigma surrounding both depression and HIV.

Our chatbot should meet adolescents living with HIV “where they are at,” providing linkage to a mental health specialist as needed but also helping adolescents living with HIV learn healthy coping strategies. Moreover, future versions of our chatbot could be adapted to address other psychosocial issues, including HIV disclosure, sex, sexuality, dating and romantic relationships, stigma, body image, and other common psychosocial issues among adolescents, and could be easily adapted for delivery in multiple languages. As with all health problems, early intervention is associated with improved outcomes both at the individual and programmatic levels. At the individual level, adolescents living with HIV who learn how to recognize depression and enact healthy coping skills may be able to stave off the escalation of depression and associated disengagement with HIV care. At the programmatic level, enhanced mental health support for adolescents living with HIV could ultimately reduce missed clinic appointments, improve ART adherence (and consequently less need for inpatient care), and ultimately lead to better HIV outcomes for this vulnerable population.

Anticipated limitations of this study include the rapid formative and chatbot development period (12 months) with participants from 1 geographic location (Lima), which may not address experiences of depression by adolescents living in rural areas. Further, the small (convenience) sample of adolescents who will test the chatbot (phase 3) will preclude tests of association. Nonetheless, we expect that some participants (including youths, their caregivers, and HIV care professionals) will have previously lived in rural areas and could share insights from those experiences. Although the sample size precludes tests for association, the data collected using a battery of validated instruments from this pilot study should provide a clear indication of the acceptability and feasibility of the chatbot to facilitate mental health screening, education, self-help, and linkage to care among adolescents living with HIV and provide the groundwork for a future, appropriately powered study to determine the efficacy of the approach.

This study will contribute to a growing body of literature on the use of chatbots in the health services delivery sector and hold promise for addressing a mental health service gap among adolescents with HIV by providing education, self-help skills, and care linkage. If our chatbot is determined to be feasible and acceptable among adolescents in this study, future iterations could be expanded beyond depression (eg, anxiety, disordered eating and body image, and HIV and mental health stigma) and disseminated beyond the single geographic area in this study (ie, to rural areas and, potentially, other countries in Latin America).

## Appendix

Multimedia Appendix 1 Peer-review report by the Collaborative Initiative for Paediatric HIV Education and Research.

## Abbreviations

ART: antiretroviral therapy
COREQ: Consolidated Criteria for Reporting Qualitative Data
IRB: institutional review board
YAB: Youth Advisory Board

## Data Availability

The data sets generated and analyzed during this study will be available from the study investigators (JTG and CC) on reasonable request.
