# Supplementary material for: Development and Pilot-Testing of an Optimized Conversational Agent or “Chatbot” for Peruvian Adolescents Living With HIV to Facilitate Mental Health Screening, Education, Self-Help, and Linkage to Care: Protocol for a Mixed Methods, Community-Engaged Study
Source: JMIR Res Protoc. 2024 May 7;13:e55559. doi: 10.2196/55559 (PMC11109861; doi:10.2196/55559)
Supplement: Multimedia Appendix 1 [file resprot_v13i1e55559_app1.pdf]

Collaborative Initiative for Paediatric HIV Education and Research (CIPHER)

Research Grant Programme

PEER REVIEW COMMENTS REPORT

**Application ID**	1128
**Applicant**	Jerome Galea
**Applicant's institution**	University of South Florida
**Country of Applicant's institution**	United States
**Project Title**	Feasibility and acceptability of chatbot-delivered depression education, self-help, and care linkage for Peruvian adolescents living with HIV.
**Application Link**	Link

Reviewer 1:

Significance

Strengths :

- Very positive impression of the proposed research It has potential for high-value contribution to patient care in HIV and more generally for a variety of physical and mental health conditions. If successful, PI plans to seek inclusion of chatbot technology into national guidelines for HIV patient care.

Investigator

Strengths :

- 1. PI's research and clinical background and experience with adolescents living with HIV 2. Long-term, established relationships with colleagues and institutions in US and Peru (study site)

Approach

Strengths :

- 1. Detailed description of process to develop, test, and implement chatbot is a strength of the proposal 2. Inclusion of study participants across adolescent age range (10-19 years) and categories of sex and gender identity

Weaknesses :

- 1. What are the plans for communication between PIs and investigators in US and Peru, besides travel and in-person meetings? 2. What are the training requirements for study personnel conducting focus groups and individual interviews with adolescents, caregivers, and health care providers 3. What are the inclusion/exclusion criteria for eligibility for participation? 4. What are plans for debriefing and follow-up treatment (if needed) since chatbot will not be finished at the end of the pilot study?

Innovation

Strengths :

- 1. Chatbot technology is being introduced in work with children with autism to improve social communication skills and with adolescents with HIV to improve medication adherence. These are relatively new applications, to my knowledge, that will be enhanced with data from the proposed study.

Environment and resources

Mentor Score :

Overall impact and scientific merit

Strengths :

- 1. A strong "yes" to all. 2. This project has the potential to make a significant contribution to treatment approaches not only for people living with HIV but for many with other chronic conditions of mental and physical health. 3. The PI plans to use data from the pilot study to support future research.

Budget : Yes, the proposed total budget for this project is realistic and reasonable.

1. I cannot evaluate the dollar amounts per line item but think the budget is reasonable in that it provides for the expenses expected for a project of this kind.
2. Are there any plans to reimburse participants for time and travel expenses incurred during study participation? The study will provide snacks for focus group and individual interviews.

Reviewer

Significance Score :

Strengths :

- Important focus on mental health care needs of ALWH in a resource limited setting, in particular those with mild to moderate depression. Nice inclusion of key stakeholders including the caregivers of ALWH. Testing an inexpensive, adaptable and potentially scalable intervention.

Weaknesses :

- Support for use of a chatbot to address mild to moderate depressive symptomatology is not very strong. While the application includes data on adolescents access to the "web" in Peru, there are no data mentioned on adolescents access to a smartphone as this is, I am assuming how the intervention would be delivered if found to be feasible/acceptable, although not stated.

Investigator Score :

Strengths :

- Assistant professor at the University of South Florida. Worked for over 15 years at Socios En Salud - the proposed location for this study in Peru. PI has 7 first author publications and 7 co-author publications. Has worked on a number of research studies.

Weaknesses :

- Nothing of note.

Approach Score :

Strengths :

- Community-engaged approach to the research. Will be working with a Youth Advisory Board. Formative work is iterative and then followed by a pilot. Grouping of the focus groups by age. Use of a theoretical framework to guide the research. Plan to collect gender identity. Appropriate timeline.

Weaknesses :

- Might be important to also separate focus groups by gender, especially for younger adolescents, as having mixed gender groups might impact how open the adolescents are in the group format and gender specific groups might also help to elicit gender specific issues that need to be addressed. Unclear who is on the YAB - number of adolescents, ages, how many report some depressive symptoms or history of depression, etc. How representative is it of the target population for the chatbot intervention? Talk about including sexual minority youth but don't mention whether they will be collecting sexual identity. Important given the role that it might play in depression. Will the 50 participants in Aim 2 only interact with the chatbot once? Seems as though to really assess the feasibility and acceptability, it should be assessed over a longer period of time. The preferred term is transgender female, not transgendered female. Given the wide age range, will these be youth that were born with HIV, those who have acquired HIV, or both? Important to capture these data as it may have

implications for depression treatment. For example, ALWH who was perinatally infected may be an orphan. Nothing on how the chatbot would handle suicidal ideation. If questions about suicidal ideation are included, then there needs to be a strong plan for how to quickly and appropriately respond. Also how the team will respond if issues around suicide are brought up during the focus groups. Nothing is included on the consent/assent process. What age can youth consent for themselves? How will parental or caregiver consent be obtained for those that cannot provide consent for themselves?

Innovation Score :

Strengths :

- Innovative approach to addressing unmet mental health care needs of ALWH

Weaknesses :

- nothing to note

Environment and resources Score :

Strengths :

- Strong collaborators (SES and the National Institute for Child Health HIV care program). The PI has a long history of collaborating with SES as Dr. Galea worked there for over 15 years. Letters of support from someone in the Peruvian Ministry of Health, the National Institute of Child Health, SES, and SES's YAB. USF will be providing one PhD student mentee at 10 hours/week.

Weaknesses :

- none

Mentor Score :

Strengths :

- Strong primary mentor (Molly Franke) whom the PI has worked with for a few years now. Strong mentoring plan with a focus on grantspersonship.

Weaknesses :

- Unclear if there is someone on the team who has experience with the use of chatbots.

Overall impact and scientific merit :

Strengths :

- Important area to focus on - unmet mental health needs of ALWH. Strong candidate and strong set of collaborators.

Weaknesses :

- A number of minor concerns with the approach, the biggest of which is a question about how long the youth will be testing the chatbot during the pilot. Seems insufficient to only have them use it once. Unclear if there is anyone on the team that has experience delivering interventions via chatbots.

Budget : No, the proposed total budget for this project is not realistic and/or reasonable.

10% effort for the PI seems very low for the research and training goals that have been laid out.

Reviewer 3:

Significance

Strengths :

- The project meets requirements for a number of CIPHER priorities: Service delivery for HIV Adolescent population Resource limited settings Assessing a mobile and user-friendly intervention to improve HIV cascade Scientific knowledge will be advanced to enable screening that is user-friendly and able to offer an improved service to many more vulnerable youth. Potential impact of this research on policies and programming: This is new and innovative technology and appropriate for the target population. If the study shows to be successful should be able to broaden to many sites and improve accessibility to care for

ALWH with mental health issues.

Weaknesses :

- Pilot study - which will require further work (that can also be a strength given the CIPHER policy)

Investigator

Strengths :

- Investigator: The PI has experience working at of the proposed study site. Collaborators: Co-investigators at the study site seem to be appropriate skills choice (psychologist, information technology, grant funding proposals) Vital component of the Youth Advisory (YAB) board of ALWH could be seen as key collaborators. The YAB are the most realistic as they represent the target participant population

Weaknesses :

- none noted

Approach

Strengths :

- Well described except that I had to educate myself what a chatbot was (giving away my generational gap). Once this was clear then all fell into place nicely and supported by the approach. Clear step-wise approach which is practicable.

Weaknesses :

- none

Innovation

Strengths :

- Yes moving towards digital world which is much user-friendly and appropriate for the target population.

Weaknesses :

- none

Environment and resources

Strengths :

- The study will take place in an existing site that is well known to PI and with collaborators who appear to have a good working relationship. Seems the study will work suing existing environment, populations and collaborations that have worked well before.

Weaknesses :

- none identified

Mentor

Strengths :

- The PI could most likely run this without too much mentoring. However as described, the Mentor will assist with grant writing for future follow on projects on the basis of this study - which is a requirement for CIPHER trainees. The mentor plan for this is appropriate and well described and has been shown to work previously.

Weaknesses :

- Does the PI really need to register for CIPHER grant to achieve the aims for personal mentorship for a larger grant? PI is already experience at conducting research.

Overall impact and scientific merit :

Strengths :

- Yes to all the questions in 7 above. If the pilot study shows that there is no improvement then that is important as well for future research to adapt and change direction. Regardless of results preliminary data is likely to lead to independent grant funding.

Weaknesses :

- none

**Budget : Yes, the proposed total budget for this project is realistic and reasonable.**
Professional transcriptions from focus group possibly underbudgeted - depends of length of meeting and professional fees in Peru
